# The Development of a Stakeholder Member Organization to Advocate for Geriatrics Education

**DOI:** 10.1093/geroni/igaa057.1801

**Published:** 2020-12-16

**Authors:** Leland Waters, Elyse Perweiler

**Affiliations:** 1 Virginia Commonwealth University, Richmond, Virginia, United States; 2 Rowan University School of Osteopathic Medicine, Stratford, New Jersey, United States

## Abstract

The National Association of Geriatric Education Centers organization was established in 1990, to promote interdisciplinary geriatric education and to provide a unified voice for Geriatric Education Centers (GECs). In 2005, the GECs voted to form two non-profit organizations due to restrictions related to lobbying activities. An umbrella organization was created, the National Association for Geriatric Education, that includes all geriatric related education programs, and maintain a lobbyist in Washington DC to protect the GECs interests. It was a pivotal time, as we had a year (2006) without federal funding that summarily dismantled the DHHS-HRSA geriatrics programs, including the entire GEC network, the geriatric fellowship program, and Geriatric Academic Career Awards. This resulted in a GEC-wide and national geriatrics movement that succeeded in restoring the geriatrics line item in the President’s budget. Our advocacy efforts not only had the line item restored, but obtained an increase in funding for geriatrics.

